# Book Review: Action and Interaction

**DOI:** 10.3389/fpsyg.2020.01598

**Published:** 2020-07-21

**Authors:** Edward Baggs

**Affiliations:** Rotman Institute of Philosophy, University of Western Ontario, London, ON, Canada

**Keywords:** theory of mind, interaction, embodied cognition, enactivism, interaction theory

It is an underappreciated fact that the way that we currently think about “theory of mind” is in substantial part thanks to Dan Dennett. Nobody was using the phrase “theory of mind”—at least not in the sense of understanding others' beliefs, and not in print—until Premack and Woodruff (1978) published their seminal study asking, Does the chimpanzee have a theory of mind? Premack and Woodruff sought to determine whether a chimpanzee could recognize the intentions of a human actor from a videotaped scene. They concluded that the chimpanzee can indeed recognize the actor's intentions. In his (insightful) commentary on this study, Dennett (1978) pointed out that Premack and Woodruff's experimental design nevertheless left open the possibility that the chimpanzee was simply using associative learning, and need not necessarily have understood the actor's intentions at all. The only way to know for sure whether the chimpanzee really does have a theory of mind, Dennett said, would be to ask whether the chimpanzee understands *false* beliefs. Dennett went on to describe an experimental setup to address this question. The false belief test that Dennett described was subsequently adapted for use by human developmental researchers, who used it to ask the same question about whether their subjects understand false beliefs, although with an important methodological adjustment (children can give a verbal, as opposed to a merely behavioral, response to a question about their beliefs). It was found, seemingly, that human children do not have a theory of mind until they are 4 years old (Wimmer and Perner, 1983), while autistic children perhaps do not have one at all (Baron-Cohen et al., 1985). The only problem is that nobody has been able to agree on what this phrase, “theory of mind,” actually refers to.

Shaun Gallagher, in his book, omits any reference to Dennett's commentary. This is a shame as it is useful to know that a philosopher helped get us into this predicament, if only to set in its proper context Gallagher's implicit claim here that it is going to take another philosopher (himself) to get us out of it.

At the heart of Gallagher's book is a chapter that lays out “the case against theory of mind.” Gallagher has examined innumerable competing accounts of ToM, all of which broadly fall into at least one of two categories: theory theory (TT) or simulation theory (ST). Gallagher usefully identifies three suppositions that all such accounts (whether TT or ST) seem to share: (1) the unobservability principle (we cannot directly perceive other minds); (2) the observational stance (we perceive others primarily from the perspective of third-person observers); (3) the supposition of universality (it is assumed that the basic cognitive mechanisms underlying behavior in *some* social situation must be implicated in *all* social situations). Gallagher wants to reject all three of these postulates. Gallagher goes on to identify a series of pitfalls for existing ToM accounts, the most serious of which seems to be a version of the frame problem—that old nemesis of artificial intelligence researchers (Gallagher here calls it “the starting problem”): how do we know when to apply our ToM mechanisms unless we have *already* understood that we are in a social situation? Gallagher's discussion of the suppositions of existing accounts of theory of mind, and of the problems faced by these accounts, is forensic and convincing.

As an alternative to TT and ST accounts Gallagher offers his own account, Interaction Theory (IT). This is based on an enactive understanding of the individual as primarily a second-person participant in ongoing activities, rather than as a detached third-person observer. The central insight is that we are rarely called upon, in real life, to answer questions from a detached observational standpoint about others' minds. Instead, we are participants in situations that already involve multiple actors. We are second-persons.

One wonders, though, whether IT really is an alternative to TT and ST. Gallagher, after all, has already rejected the very question that ToM accounts are supposedly trying to address. ToM starts with the question: How do we *know* other minds. The assumption is that we need to have our own private understanding of other minds before we can engage in behavior with others. But Gallagher has rejected that this is an appropriate starting assumption. On Gallagher's account, what comes first is the action, not the understanding. We start out by simply acting in a setting that is populated with other actors—we start out as participants (Reddy and Morris, 2004). Only later, through our learning of language and narrative practices, do we begin to engage in folk psychological ways of thinking about one another *as* persons. Moreover, Gallagher argued in his previous book (Gallagher, 2017) that enactivism itself (which includes IT) is less a positive program of scientific research, and more a “philosophy of nature”: it is a way of thinking about mind and behavior that situates these things within their proper holistic context. This kind of project, however, by definition does not lend itself naturally to supporting a program of laboratory-based scientific research (Barrett, 2019). IT, then, is less a competitor theory to TT and ST accounts than a wholesale rejection of the very framework of ToM thinking.

In the final part of the book Gallagher turns to a critique of political institutions. Gallagher suggests that, from an embodied, enactivist standpoint, liberal theories of justice, such as that of John Rawls, are fundamentally misguided. Just as we don't need a theory of mind to interact with our mothers, so we don't need a theory of justice to recognize whether or not the world around us is structured in a just way.

Gallagher's book demonstrates, ultimately, that there is no such thing as an ideologically neutral way of doing cognitive science. The ToM framework is built on an ideological foundation of rational individualism. This was never a good place to start when asking questions about how chimpanzees see the world, and it was no more useful when the logic was transferred to humans. Gallagher is right: it is time that we moved beyond ToM.

## Author Contributions

The author confirms being the sole contributor of this work and has approved it for publication.

## Conflict of Interest

The author declares that the research was conducted in the absence of any commercial or financial relationships that could be construed as a potential conflict of interest.
